# Utilization of virtual low-keV monoenergetic images generated using dual-layer spectral detector computed tomography for the assessment of peritoneal seeding from ovarian cancer

**DOI:** 10.1097/MD.0000000000020444

**Published:** 2020-06-05

**Authors:** Taek Min Kim, Sang Youn Kim, Jeong Yeon Cho, Seung Hyup Kim, Min Hoan Moon

**Affiliations:** a Department of Radiology, Seoul National University Hospital; b Department of Radiology, Seoul National University College of Medicine; c Institute of Radiation Medicine and Kidney Research Institute, Seoul National University Medical Research Center; d Department of Radiology, SMG-SNU Boramae Medical Center, Seoul National University College of Medicine, Seoul, Korea.

**Keywords:** dual-energy CT, dual-layer spectral detector CT, ovarian cancer, peritoneal implant, virtual monoenergetic image

## Abstract

This study aimed to compare the quality of virtual low-keV monoenergetic images vs conventional images reconstructed from dual-layer spectral detector computed tomography (SDCT) for the detection of peritoneal implants of ovarian cancer.

Fifty ovarian cancer patients who underwent abdominopelvic SDCT scans were included in this retrospective study. Virtual monoenergetic images at 40 (VMI_40_) and 50 keV (VMI_50_), and two conventional images were reconstructed using filtered back projection (FBP) and iterative model reconstruction (IMR) protocols. The mean attenuation of the peritoneal implant, signal-to-noise ratio (SNR), contrast-to-noise ratio relative to ascites (CNR_A_) and adjacent reference tissues (e.g., bowel wall, hepatic, or splenic parenchyma [CNR_B_]) were calculated and compared using paired *t* tests. Qualitative image analysis regarding overall image quality, image noise, image blurring, lesion conspicuity, was performed by two radiologists. A subgroup analysis according to the peritoneal implant region was also conducted.

VMI_40_ yielded significantly higher mean attenuation (183.35) of SNR and CNR values (SNR 11.69, CNR_A_ 7.39, CNR_B_ 2.68), compared to VMI_50_, IR, and FBP images (*P* < .001). The mean attenuation (129.65), SNR and CNR values (SNR 9.37, CNR_A_ 5.72, CNR_B_ 2.02) of VMI_50_ were also significantly higher than those of IR and FBP images (*P* < .001). In the subgroup analysis, all values were significantly higher on VMI_40_ regardless of the peritoneal implant region (*P* < .05). In both readers, overall image quality and image blurring showed highest score in VMI_50_, while image noise and lesion conspicuity showed best score in IMR and VMI_40_ respectively. Inter-reader agreements are moderate to almost perfect in every parameter.

The low-keV VMIs improved both quantitative assessment and lesion conspicuity of peritoneal implants from ovarian cancer compared to conventional images.

## 1 Introduction

Approximately 90% of ovarian cancers are epithelial ovarian cancers. It most frequently disseminates via the transcoelomic route except for direct extension, with about 70% of patients having peritoneal metastases at staging laparotomy.^[1]^ Cytoreductive surgery is considered for epithelial ovarian cancer at the time of initial treatment and recurrence, and it has been established that improved survival after surgery is associated with minimal-volume residual disease.^[2]^ So the accurate imaging-based detection of peritoneal metastases is important to the staging and follow-up of ovarian cancer. Currently, computed tomography (CT) is considered the best imaging modality for the evaluation of patients with known or suspected peritoneal metastases.^[3,4]^ Recent study of peritoneal implants from ovarian tumors have indicated a sensitivity of 83% and a specificity of 86% for the correlation of pathologic and CT diagnoses.^[5]^ The sensitivity decreased to 25% to 50% for detection of implants less than 1 cm in size.^[6]^

Recent technical developments in the field of clinical radiology have led to a re-emergence of dual-energy CT (DECT).^[7]^ Dual-layer spectral detector CT (SDCT), the most recently developed dual-energy technique, uses a single polychromatic x-ray source and detects the photons of lower energies in the surface and of higher energies in the layer below.^[8]^ This allows dual-energy analysis to be performed on every data set acquired, which enables to generate spectral images such as virtual monoenergetic image (VMI). In several studies, low-energy VMIs generated via SDCT have yielded high levels of contrast between iodine-enhanced lesions and adjacent tissues.^[9,10]^ Because the peritoneal implants enhance with intravenous contrast material, we presumed low-energy VMIs in SDCT may be helpful for assessment of peritoneal seeding, even in small lesion. The present study aimed to compare the image quality of low-keV VMIs with conventional images reconstructed using SDCT to address the challenges associated with the assessment of peritoneal implants of ovarian cancer.

## 2 Materials and methods

### 2.1 Patient selection

This retrospective, single-center study was approved by Institutional Review Board of Seoul National University Hospital (IRB No: 1805-066-946). The requirement for written informed consent was waived due to the mandatory nature of abdominopelvic CT examination during routine clinical practice.

We retrospectively evaluated a total of 50 abdominopelvic CT scans from ovarian cancer patients that were obtained at our institution using a standard DECT protocol during follow-ups for ovarian cancer between February and July 2017. The 50 images were obtained from 50 female patients with a mean age of 58.02 ± 12.19 years. All patients met the following eligibility criteria:

1. pathological diagnosis of ovarian cancer,
2. receipt of abdominopelvic SDCT scans with available virtual monoenergetic reconstructions,
3. previous abdominopelvic CT scans available for comparison, and
4. available clinical data, including laboratory findings such as the serum carbohydrate antigen (CA)-125 level.

For all patients, the electronic medical records regarding pathologic findings, laboratory findings, operative history, body mass index and clinical course were reviewed. The demographics of the study population are summarized in Table 1.

**Table 1:**
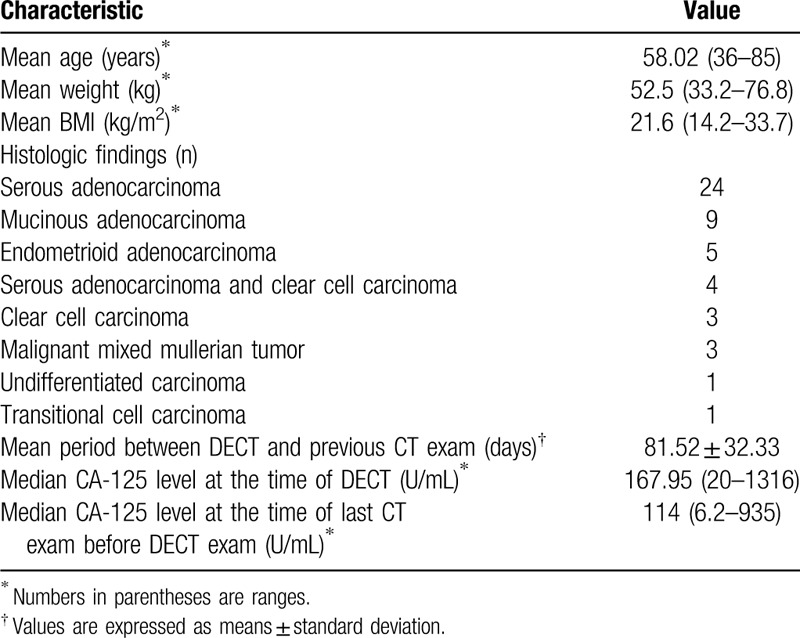
Demographics of the study population.

### 2.2 Image acquisition

Imaging data were acquired on a 128-channel SDCT (IQON spectral CT; Philips Healthcare, Cleveland, OH) with a tube voltage of 120-kVp under automated tube current modulation. The acquisition parameters were as follows: rotation time: 0.33 s; detector collimation: 64 × 0.625 mm; pitch: 0.891; matrix: 512 × 512. All axial images were reconstructed with a slice thickness of 3 mm and a slice increment with 3 mm. The scan range was from the top of the liver to the pubic symphysis. Iodinated contrast media at a concentration of 350 mgI/mL (iohexol; Bonorex 350, Central Medical Service, Seoul, South Korea) was administered into a peripheral vein in the upper extremity via an automatic power injector at a total dosage of 1.6 ml/kg over 30 s. Biphasic post-contrast imaging was performed to include the arterial (30-s delay after the aortic signal reached 100 HU using the bolus tracking method) and delayed (fixed 3-min delay) phases, as indicated clinically. Because arterial phase did not cover upper abdomen in this protocol, only delayed phase images were subjected to analysis.

For delayed phase images, conventional 120-kVp images were reconstructed using filtered back projection (FBP) and iterative reconstruction algorithms, iterative model reconstruction (IMR). VMIs were reconstructed at 40 (VMI_40_) and 50 keV (VMI_50_) retrospectively. For VMI, iterative reconstruction algorithm, IMR was also used.

Previous CT examinations for comparison were taken with same protocol, CT parameters, type and quantity of contrast agent as above, and reconstructed to the same conventional FBP and IMR images.

### 2.3 Quantitative image analysis

The VMI_40_, VMI_50_, FBP, and IMR images were retrospectively subjected to an objective image analysis using a commercially available PACS workstation (Infinitt, Infinitt Healthcare, Seoul, Korea). Upon achieving consensus, two radiologists obtained mean and standard deviation (SD) CT attenuations (in Hounsfield units; HU) for the peritoneal implant, adjacent tissue parenchyma (bowel wall, liver or splenic parenchyma), and ascites by manually placing circular ROIs at the same image level for every image set. Peritoneal implants were defined as nodular, plaque-like or infiltrative soft tissue lesions in peritoneal fat or on the serosal surface with parietal peritoneal thickening or enhancement and, most importantly, an unequivocal size increase or new appearance since previous exam that exhibited the same trend as the increase of serum CA-125 level. The size change of peritoneal implants were assessed in IMR images at delayed phase side by side.

**Figure 1:**
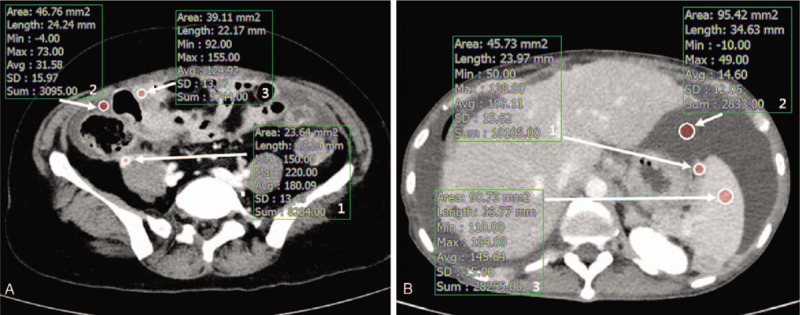
Obtaining ROIs for quantitative image analysis. The average CT attenuation and standard deviation (SD) of the peritoneal implant and were obtained from a single ROI drawn in the most enhancing solid portion of the lesion (Box 1). The adjacent ascites were measured for reference tissue (Box 2). (A) The virtual monoenergetic image at 40 keV in 52-year-old ovarian cancer patient. For cases in which the peritoneal implant was located near the bowel, mean attenuation and SD of the adjacent small or large bowel wall was additionally obtained (Box 3). (B) The virtual monoenergetic image at 40 keV in 62-year-old ovarian cancer patient. For cases in which the peritoneal implant was located in the surface of liver or spleen, the mean attenuation and SD of the adjacent liver or splenic parenchyma was additionally obtained (Box 3).

The single ROI was drawn in the most enhancing solid portion of the peritoneal implant (mean size: 34.5 mm^2^). The lesions which were under 0.3 cm in size were excluded for evaluation for the accuracy of measurement. If the peritoneal implant was located near the bowel, another ROI was drawn in the most homogeneous area of adjacent small or large bowel wall (mean size: 36.2 mm^2^) (Fig. 1A). In case of the peritoneal implant was located in the surface of liver or spleen, ROI was drawn in the adjacent liver or splenic parenchyma (mean size: 85.5 mm^2^) (Fig. 1B). Large vessels, bile duct, areas of necrosis or calcification, and focal lesions were carefully avoided. Additionally, the ROI was also drawn in the most nearby ascites for each peritoneal implant (mean size: 87.2 mm^2^). Image sets from the same examination, including the sizes, shapes, and positions of the ROIs, were kept constant at the workstation by using the copy and paste function. The peritoneal cavity was classified into seven regions: perihepatic space, perisplenic space, right paracolic gutter, left paracolic gutter, small bowel mesentery, sigmoid mesocolon, and posterior-cul-de-sac (PCDS). Each peritoneal implant was evaluated according to these regions.

For each image set, the signal-to-noise ratio (SNR) of the peritoneal implant and contrast-to-noise ratio relative to the ascites (CNR_A_) and adjacent reference tissue such as bowel wall, liver or splenic parenchyma (CNR_B_) were calculated respectively using the following equations:  
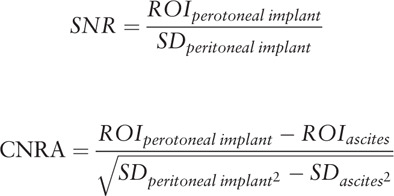



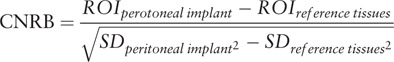


### 2.4 Qualitative image analysis

The subjective image analysis was performed by two experienced radiologists (S.Y.K. and T.M.K. with 15 and 5 years of genitourinary imaging experience, respectively). The reviewers were asked to evaluate four qualitative features in each image sets using 5-point Likert scale. The features include overall image quality ranging from 1, nondiagnostic to 5, excellent, subjective image noise ranging from 1, extensive image noise to 5, absence of noise, imaging blurring ranging from 1, severe blurring, edge definition very poor to 5, no blurring, edges well defined, and conspicuity of peritoneal seeding lesion as follows:

1. poor lesion delineation with insufficient contrast to adjacent tissue
2. difficult lesion delineation with subtle contrast to adjacent tissue
3. intermediate lesion delineation with moderate contrast to adjacent tissue
4. sufficient lesion delineation with clear contrast enhancement
5. excellent lesion delineation with strong contrast enhancement

All image series were assessed in random order to avoid potential bias. The reviewers were blinded to the applied reconstruction technique and VMI energy level.

### 2.5 Radiation dose evaluation

The volume CT dose index (CTDIvol) and dose–length product (DLP) were obtained by reviewing the dose reports from each examination. The CTDIvol was determined with reference to a 32-cm phantom.

### 2.6 Statistical analysis

A one-way analysis of variance (ANOVA) with Dunnett post hoc analysis was used to compare CT attenuation in the peritoneal implant, SNR, CNR_A_, CNR_B_, and qualitative image parameters among VMI_40_, VMI_50_, IMR, and FBP images. Subgroup analysis was performed according to the region in which the peritoneal implant was located. A *P* value <.05 was considered to indicate statistical significance. Ratios of improvement in SNR and CNR values were compared between VMI_40_ and conventional FBP and IMR images according to the region. Interreader variability was calculated by using weighted κ statistics and interpreted as follows: 0.21–0.40, fair; 0.41–0.60, moderate; 0.61–0.80, substantial; and 0.81–1.00, almost perfect. A commercially available software package (SPSS 21.0 for Windows; IBM, Inc, Armonk, NY) was employed for the statistical analyses.

## 3 Results

### 3.1 Quantitative image analysis

The quantitative measurements of image quality in VMI_40_, VMI50, FBP, and IMR images are summarized in Table 2. The average CT attenuation, SNR, CNR_A_, and CNR_B_ of the peritoneal implant was significantly higher in VMI_40_ (mean ± SD, 183.35 ± 48.51, 11.69 ± 4.87, 7.39 ± 2.98, 2.68 ± 1.96, respectively) than in VMI_50_ (129.65 ± 33.25, 9.37 ± 3.56, 5.72 ± 2.23, 2.02 ± 1.50), IMR (83.04 ± 19.83, 8.62 ± 3.85, 5.10 ± 2.12, 1.72 ± 1.33) and FBP images (83.33 ± 19.81, 5.50 ± 2.12, 3.16 ± 1.20, 1.10 ± 0.86) (all, *P* < .05). Notably, VMI_50_ also yielded significantly higher average CT attenuation, SNR, CNR_A_ and CNR_B_ values compared to IMR and FBP images (all, *P* < .05).

**Table 2:**
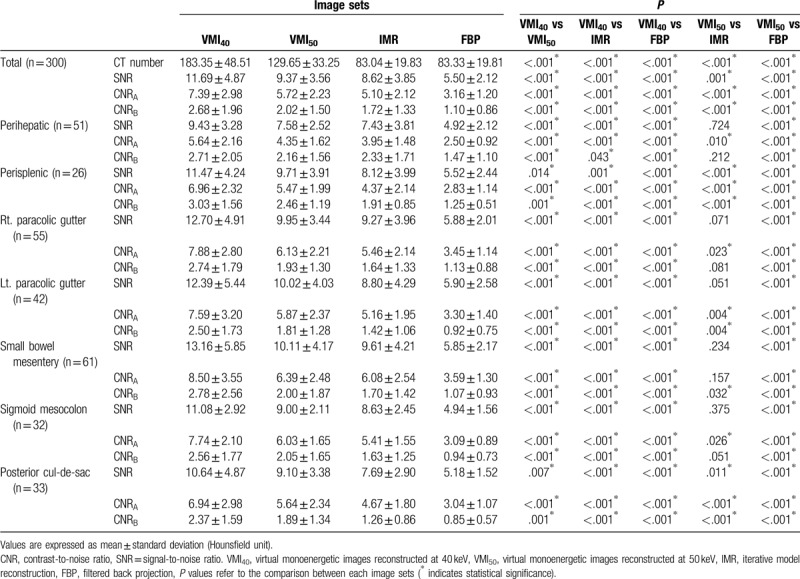
Comparison of CT number, SNR, CNR_A_, and CNR_B_ between virtual monoenergetic images at 40-keV, 50-keV and conventional 120-kVp images with IMR and FBP reconstruction technique with subgroup analysis according to the location of peritoneal implants.

### 3.2 Region-based quantitative analysis of monoenergetic image quality

Table 2 summarizes the results of a subgroup analysis according to peritoneal implant location. In all locations, VMI_40_ had higher SNR, CNR_A_, and CNR_B_ values relative to the other image sets (all, *P* < .05). Furthermore, VMI_50_ had significantly higher SNR, CNR_A_, and CNR_B_ values relative to FBP images, regardless of location (all, *P* < .05). By contrast, although the overall quantitative image quality was significantly higher with VMI_50_ than with IMR, these image sets did not differ significantly with respect to the SNR and CNR_B_ in the perihepatic (*P* = .724 and *P* = .212, respectively), the right paracolic gutter (*P* = .071 and *P* = .081), the sigmoid mesocolon (*P* = .375 and *P* = .051), the SNR and CNR_A_ in the small bowel mesentery (*P* = .234 and *P* = .157) or the SNR in left paracolic gutter (*P* = .051).

**Figure 2:**
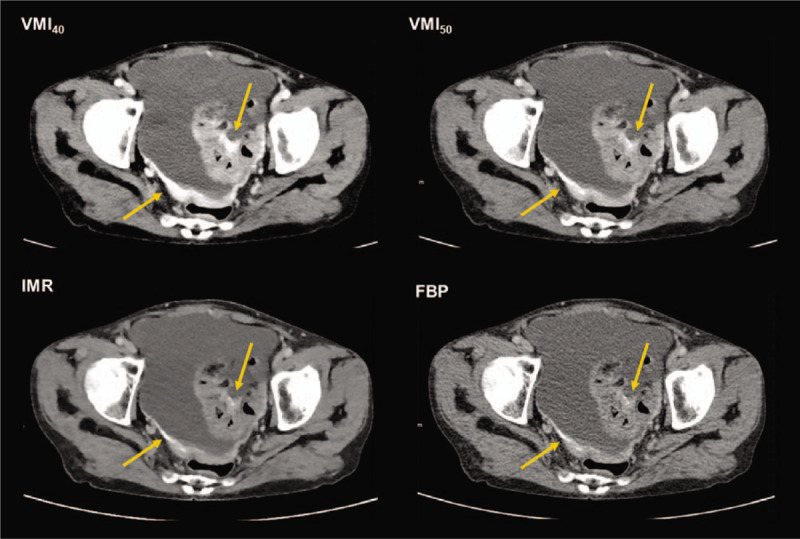
Fifty-seven years old female patient with peritoneal enhancement and thickening in the pelvic peritoneum and sigmoid serosa (arrows). More prominent enhancement is observed in a virtual monoenergetic image at 40 keV (VMI_40_), compared to VMI_50_ and conventional images reconstructed using iterative model reconstruction (IMR) and filtered back projection (FBP).

**Figure 3:**
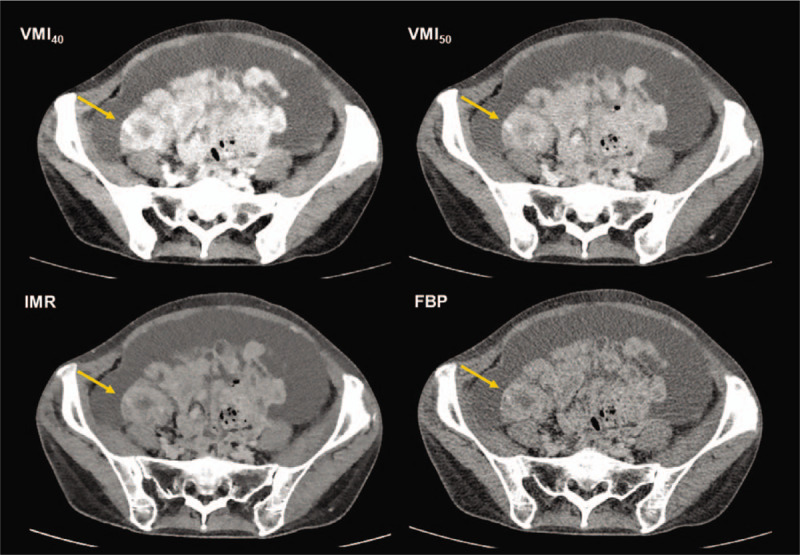
Sixty-two years old female patient with a seeding lesion in the small bowel wall (arrow). The seeding lesion is most strongly enhanced in the virtual monoenergetic image at 40 keV (VMI_40_), compared to VMI_50_ and conventional images reconstructed using iterative model reconstruction (IMR) and filtered back projection (FBP).

**Figure 4:**
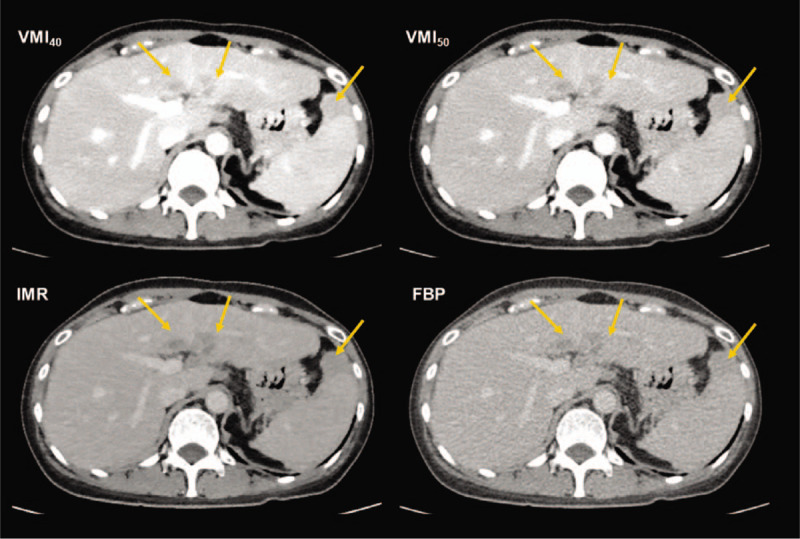
Sixty-nine years old female patient with perihepatic and perisplenic seeding lesions (arrows). A greater contrast difference was observed in the virtual monoenergetic image at 40 keV (VMI40), compared to VMI50 and conventional images reconstructed using iterative model reconstruction (IMR) and filtered back projection (FBP).

The ratios of improvement of quantitative image quality between VMI_40_ and conventional images using FBP and IMR techniques are summarized in Table 3 (Figs. 2–4).

**Table 3:**
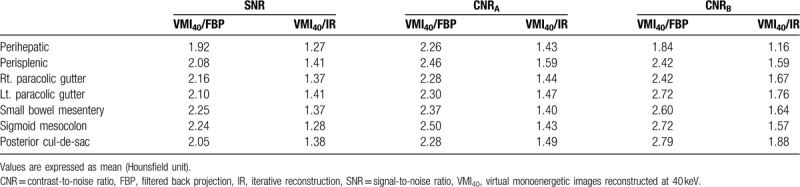
The ratios of improvement of SNR, CNR_A_, and CNR_B_ between virtual monoenergetic images at 40-keV and conventional 120-kVp images with IR and FBP reconstruction technique according to the location of peritoneal implants.

### 3.3 Qualitative image analysis

In both readers, the score of overall image quality and image blurring were highest in VMI_50_, followed by IMR images. VMI_40_ obtained significantly lower score than VMI_50_ or IMR images (all, *P* < .05). In case of image noise, IMR obtained the highest score, followed by VMI_50_ in both readers. The score of subjective image noise in VMI_40_ was significantly lower than that of IMR or VMI_50_ images (all, *P* < .05). However, the conspicuity of the peritoneal seeding showed highest score in VMI_40_, followed by VMI_50_, IMR, and FBP (all, *P* < .05) in both readers. Interreader agreements are moderate to almost perfect in all parameters (0.472–0.931). The scores of qualitative image analysis were summarized in Table 4.

**Table 4:**
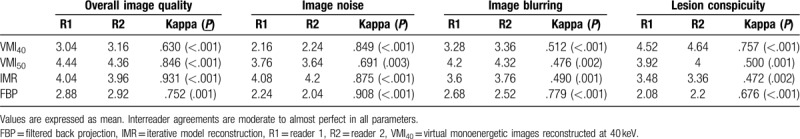
The qualitative image analysis of virtual monoenergetic images at 40-keV, 50-keV, and conventional 120-kVp images with IMR and FBP reconstruction technique.

### 3.4 Radiation dose evaluation

The average CTDIvol and DLP for abdominopelvic SDCT in delayed phase were 5.71 mGy (range, 4.5–10.9 mGy) and 315.45 mGy·cm (range, 200.3–625.1 mGy·cm).

## 4 Discussion

This study aimed to evaluate the quantitative and qualitative image parameters of VMIs generated using abdominopelvic CT data obtained from a SDCT. When detecting peritoneal implants, it is especially important to maximize the CNR and SNR during the 3-minute delayed phase because the difference in contrast between these implants and the adjacent tissues will be less obvious than in earlier contrast-enhanced phases. We found that VMI at low-energy levels yielded significantly higher CNR, SNR values and superior lesion conspicuity of peritoneal implants, and furthermore, VMI_50_ achieved higher score in overall image quality, image blurring than IMR and FBP images obtained using same SDCT.

In this study, we used a SDCT, which is a third mechanism for acquiring DECT projection data after dual-source technique and rapid kVp switching technique. It uses a single high tube potential beam and layered scintillation detectors, in which the top layer selectively absorbs low-energy photons and the bottom layer absorbs high-energy photons. An advantage to this approach is that the low- and high-energy data sets are acquired simultaneously, and the data from the inner and outer detector layers are recorded at all times.^[8]^ It facilitates the use of anti-correlated noise suppression, particularly available for detector-based dual-energy CT systems.^[11]^ Importantly, this capability allows the use of monoenergetic images at the lowest possible keV level (40 keV) for diagnostic imaging and could potentially enhance vascular contrast or improve lesion conspicuity. In a recent study, low noise levels were observed in VMI obtained in both phantom and patient experiments via a detector-based spectral CT scan, across energy levels ranging from 40 to 200 keV.^[12]^ Furthermore, combination of the projection data from upper and lower detector layer from a SDCT acquisition always offers a true conventional image data in addition to the dual-energy data, which may serve as a standard of reference while tube-based DECT systems have to depend on blended images as an alternate.^[13]^

Several previous studies demonstrated the advantages of low-keV VMI derived from SDCT. It has been reported that using low-keV VMI can improve image quality in the chest, cranial vessels and arteries in various regions.^[13–15]^ Moreover, Lee et al observed the best CNR values with 40-keV monoenergetic images and demonstrated that this option improved the diagnostic performance for active Crohn's disease when using SDCT.^[16]^ Consistent with those studies, our findings suggest that this new detector technique can quantitatively and qualitatively improve the image quality and could potentially increase the detectability of peritoneal seeding lesions. We further note that our average CTDIvol and DLP values for abdominopelvic SDCT in the delayed phase (5.71 mGy and 315.45 mGy·cm) were lower than those calculated for single energy abdominal CT (9.2 mGy and 366.8 mGy·cm) and comparable to those obtained during third-generation dual-source dual-energy abdominal CT (7.8 mGy and 310.3 mGy·cm) in a recent study.^[17]^ Since post-processing can be performed at any time, even for monophasic CT, this technique could potentially reduce the risks associated with repeated radiation exposure.

Although the CNR and SNR are often used as quantitative parameters of image quality, the absolute noise level must also be considered. While in our study we achieved the highest CNR and SNR with VMI_40_, the scores of overall image quality and subjective image noise were relatively low. A recent study demonstrated VMI_40_ images improves the detection of peritoneal metastatic deposits in dual-source dual-energy CT, and recommended not to solely interpret VMI_40_ images because of significant increase in image noise.^[18]^ However, VMI_50_ image in our study showed significantly superior quantitative image parameters and also achieved higher qualitative image qualities compared to those of IMR and FBP images. In other words, reading VMI_40_ for maximize lesion contrast in addition to conventional images or using VMI_50_ images instead of conventional images could both improve the assessment of peritoneal seeding from ovarian cancer.

The CT sensitivity and specificity according to region of peritoneal metastases were variable in recent meta-analysis.^5^ In our region-based subgroup analysis, absolute values of SNR and CNR were highest in VMI_40_ regardless of the location. The absolute values of SNR and CNR in IMR and FBP images were relatively lower in the perihepatic and perisplenic space, compared to other regions. Accordingly, it may be difficult to detect peritoneal implants in those spaces when using conventional images, whereas the use of low-keV monoenergetic images could improve the diagnostic performance of imaging for the detection of peritoneal seeding lesions. We further note that the ratios of improvement for SNR, CNR_A_, and CNR_B_ with VMI_40_ vs FBP were relatively higher in the small bowel mesentery, sigmoid mesocolon and PCDS, respectively. In case of VMI_40_ vs IMR, the highest ratios of improvement for SNR and CNR_A_ were showed in the perisplenic space, and CNR_B_ in PCDS. Therefore, we would expect improvements in diagnostic performance when using low-keV VMIs to detect peritoneal implants, especially in those spaces.

Despite the strengths of our study, we must discuss several limitations. First, this was a retrospective study performed at a single-center with a relatively small population, which may have introduced significant selection bias. Second, the evaluated peritoneal implants were not pathologically confirmed. But, we minimized this limitation by defining the peritoneal implants as peritoneal lesions that showed unequivocal increase in size or newly appear in correlation with increase of serum level of CA-125, which is a very sensitive indicator of the tumor burden. Third, the diagnostic accuracy of the peritoneal implant in low-keV VMI was not evaluated in this study. Additionally, because most of the patients showed disease progression due to the definition of peritoneal implants in our study, evaluation of the difference of staging based on the Response Evaluation Criteria in Solid Tumors (RECIST) criteria between low-keV VMIs and conventional images was impossible. Fourth, even though the reviewers were blinded to the applied image sets, identification of the reconstruction technique was possible via the textural characteristics of the images. Furthermore, the reviewers were aware that only patients with peritoneal seeding were included in this study. This might influence the results of qualitative image analysis. Lastly, the VMIs above 50 keV were not included because this study aimed to evaluate utility of low-keV VMIs in enhancing peritoneal implant due to higher photoelectric attenuation as the energies approach K-edge iodine. However, the utility of the higher energy level for assessment of peritoneal seeding should be revealed in further studies.

In conclusion, we found that the low-keV VMIs improved both quantitative and qualitative image quality for detecting peritoneal implants of ovarian cancer. Therefore, low-keV VMIs from abdominopelvic SDCT will provide additional value for the assessment of peritoneal seeding in ovarian cancer patients.

## Acknowledgments

All the authors contributed to the work described in the paper and all take responsibility for it.

## Author contributions

**Conceptualization**: Taek Min Kim, Sang Youn Kim, Jeong Yeon Cho, Seung Hyup Kim, Min Hoan Moon.

**Data curation**: Taek Min Kim, Sang Youn Kim.

**Formal analysis**: Taek Min Kim, Sang Youn Kim.

**Investigation**: Taek Min Kim, Sang Youn Kim.

**Methodology**: Taek Min Kim, Sang Youn Kim, Jeong Yeon Cho, Seung Hyup Kim, Min Hoan Moon.

**Project administration**: Taek Min Kim, Sang Youn Kim, Jeong Yeon Cho, Seung Hyup Kim, Min Hoan Moon.

**Software**: Taek Min Kim.

**Supervision**: Jeong Yeon Cho, Seung Hyup Kim, Min Hoan Moon.

**Validation**: Taek Min Kim.

**Visualization**: Taek Min Kim.

**Writing – original draft**: Taek Min Kim.

**Writing – review & editing**: Taek Min Kim, Sang Youn Kim, Jeong Yeon Cho, Seung Hyup Kim, Min Hoan Moon.
